# IgE Nonresponders in Basophil Activation Tests: A Narrative Review From Mechanistic Insights to Clinical Implications and Future Directions

**DOI:** 10.1155/jimr/2022127

**Published:** 2025-12-28

**Authors:** Bernadette Eberlein, Tilo Biedermann

**Affiliations:** ^1^ Department of Dermatology and Allergy Biederstein, School of Medicine and Health, TUM University Hospital Rechts der Isar, Technical University of Munich, Biedersteiner Straße 29, D-80802, Munich, Germany, tum.de

**Keywords:** basophil activation test, CD203c+ basophils, CD63+ basophils, Fc*ε*RI cross-linking, interleukin-3, Lyn protein, nonresponders, Syk protein

## Abstract

It has been known for about 50 years that a proportion of human basophils does not release histamine or leukotrienes or upregulates activation markers in response to IgE‐dependent stimuli. This group of so‐called nonresponders with unresponsive basophils is found in up to 20% of tests conducted. There seems to be no relation to clinical symptoms. The nonresponder status is not defined in a standardized manner, but basophils are often considered nonresponsive if they release less than 5% to 10% of their histamine content or show activation of less than or equal to 10% CD63+ basophils after Fc*ε*RI cross‐linking. Results from nonresponder patients should be regarded as false negatives. Different genetic factors between reactive and anergic basophils appear to play a minor role because various other factors allow a conversion from the nonresponder status to the responder status in one individual (IL‐3, low‐Na^+^ medium, natural changes over time, etc.). In most studies reduced levels of Lyn and Syk proteins in nonreleasers were observed. Therefore, it was assumed that translational or posttranslational regulatory mechanisms (proteasomal degradation), especially specific to the Lyn and Syk levels, are responsible for the nonresponder status. Further biochemical studies, along with mechanistic experiments and multiomics approaches, should be conducted to clarify IgE unresponsiveness.

## 1. Introduction

Basophils are effector cells in immediate‐type allergic reactions, and the clinical impact of basophil activation tests (BATs) is due to the unique ability of these cells to degranulate upon cross‐linking of the specific IgE (sIgE) bound on the membrane‐bound high‐affinity IgE receptor (Fc*ε*RI) [1]. Cellular in vitro test systems for immediate‐type allergy diagnostics utilize the detection of mediators (histamine or leukotrienes) or surface markers (CD203c, CD63; determined by flow cytometry) that are measurable upon successful basophil activation in enriched blood leukocytes or whole blood after incubation with allergens or other triggers. The most commonly used basophil activation marker, CD63, which is a component of granule membranes, is not specific to basophils and is also expressed on other blood cells. Therefore, an additional marker is required to identify basophils. Possible identification markers include anti‐CCR3, anti‐IgE, anti‐CRTH2 (excluding CD3‐positive cells), CD203c, or anti‐CD123 (excluding HLA‐DR‐positive cells). CD203c is a basophil‐specific marker and is expressed constitutively. Both CD203c and CD63 markers are upregulated after IgE receptor aggregation, but they have partially different metabolic pathways and follow different kinetics. Interleukin‐3 potentiates allergen‐induced CD63 expression without itself upregulating CD63, while it also increases CD203c expression even without allergen (referred to as the “priming” marker). Results of BATs are usually reported as a percentage of activated basophils. Cut‐off values are specified for individual allergens and controls in commercially available tests; otherwise, these must be calculated using receiver operator characteristic (ROC) curves [1].

It is known that the extent of basophil activation is individual and varies. This concept of what used to be called basophil releasability has been discussed by Lichtenstein and others since the mid‐70s [2]. It implied that there is an intrinsic property of human basophils that determines mediator release but which may be completely absent. Perhaps one of the first publications on these nonresponsive basophils in a highly pollen‐sensitive individual was an abstract presented at the annual meeting of the American Academy of Allergy in San Diego in 1975 [3]. In a larger group of patients (*n* = 93) with untreated seasonal ragweed rhinitis, strong skin sensitivity and positive sIgE, 13% of patients released insignificant amounts of histamine from their leukocytes when challenged with ragweed allergen [4]. In this manuscript the main focus is an IgE‐dependent nonresponse, which is distinguished from a complete nonresponse, that occurs in response to both IgE‐dependent and IgE‐independent stimuli and usually indicates a fundamental error or problem in the test procedure. The pathomechanisms of this phenomenon occurring in basophils have been investigated. However, the definitions of nonresponsiveness vary among studies, and there is no standardized nomenclature. This overview is intended to summarize the current state of knowledge.

## 2. Nonresponders in the Context of Cellular Tests

### 2.1. Definition and Criteria of Nonresponders

One of the first publications regarding nonresponders defined them as individuals with ≤14% histamine release from leukocytes of patients with untreated ragweed rhinitis (*n* = 93) when challenged with ragweed allergens [4]. In a review, others defined them as individuals with <10% of their histamine content after Fc*ε*RI cross‐linking [5]. In subsequent manuscripts the nonresponder phenotype refers to donor basophils (*n* = 83) that do not respond to IgE‐dependent stimulation, defined by the inability to release more than 5% histamine above spontaneous release [6]. A very stringent definition is release stimulated by a range of anti‐IgE antibody (AB) concentrations not statistically different from unstimulated release (<1%) [7]. Another group applied statistical methods to their dataset of 68 controls and 61 asthmatic subjects, defining 12.7% histamine release as the threshold between releasers and nonreleasers [8]. Some authors also considered individuals as poor responders if <20% histamine was released or if histamine release fell within the lower quintile of responsiveness [9].

**Figure 1:**
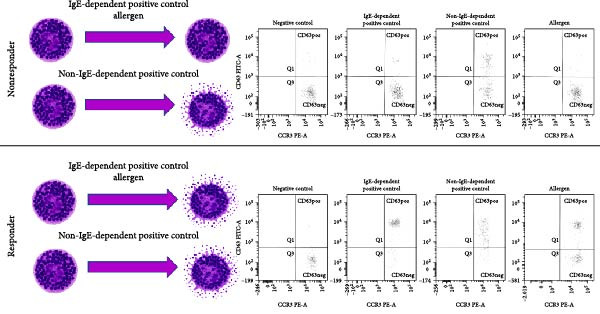
Schematic (left) and flowcytometric (right) illustration of a nonresponder and a responder in the basophil activation test. Nonresponder: IgE‐dependent stimuli (FcεRI, anti‐IgE, allergens) do not induce degranulation (lack of CD63 expression). Non‐IgE‐dependent stimulus (fMLP) leads to degranulation with CD63 upregulation. Responder: all stimuli induce degranulation and CD63 upregulation. CCR3 was used as a basophil identification marker and CD63 as a basophil activation marker in flow cytometry.

In general, basophils of such nonresponders also fail to induce CD63 and CD203c upregulation (as determined by BAT assays), generate leukotrienes, or produce cytokines in response to IgE‐dependent stimulation [10–12]. Other authors investigated 46 food‐allergic children and 29 controls, defining the nonresponder status as anti‐Fc*ε*RI‐induced CD63 expression of <10% [13]. Additionally, in commercially available BATs utilizing IgE‐dependent (anti‐Fc*ε*RI mAB or anti‐IgE) and IgE‐independent (fMLP) positive controls, a patient is considered an IgE nonresponder if the control with anti‐Fc*ε*RI mAB shows an activation ≤10% CD63+ basophils and the fMLP control shows an activation >10% CD63+ basophils [11] (Figure 1). In a study from Singapore [14] involving 476 individuals, an individual was defined as a classic “nonresponder” if less than 38% of basophils expressed CD63 after allergen stimulation but were still capable of degranulation after anti‐Fc*ε*RI stimulation. This definition does not align with a negative result in BAT, typically defined as CD63 upregulation lower than 5%, nor does it match the common definition of a nonresponder, who fails to respond to IgE‐dependent stimuli. The authors of this study used the unusual term “anergy” to describe these individuals, who lacked a response to anti‐Fc*ε*RI. This term should not be confused with the downregulation of Fc*ε*RI that can occur after allergen stimulation in vitro, potentially affecting basophils’ ability to respond to a subsequent challenge (postactivation exhaustion).

For leukotrienes a net stimulation control yield (after subtraction of background) of at least 200 pg/mL should be expected using the IgE‐dependent stimulation control. Therefore, values below this cut‐off may indicate nonresponders [15].

In summary, there is no uniform definition of nonresponder status, although the frequently used definition “IgE‐dependent stimulus activates ≤10% CD63+ basophils” appears to be appropriate in clinical practice.

### 2.2. Prevalence and Relation to Clinical Symptoms

It is generally believed that 6% to 20% (sometimes up to 28%) of individuals (depending on the cut‐off point) are nonresponders in terms of histamine and leukotriene release, as well as basophil activation, to an IgE cross‐linking stimulus [1, 8, 16, 17]. This phenotype has been observed in different ethnic groups and in both individuals experiencing allergic symptoms and healthy controls [6, 8, 18]. An analysis of house dust mite allergic patients suggested that anergic individuals (with the critically viewed definition of having <38% CD63+ basophils to anti‐Fc*ε*RI) are less likely to develop atopy [14].

### 2.3. Implications of Nonresponsiveness for Test Performance

Results from nonresponder patients should be considered as false negatives when evaluating test performance. No conclusions regarding allergen‐induced responses can be drawn. Nonresponders can experience allergic symptoms and have positive skin prick test (SPT) with relevant allergens [1]. In the EAACI task force position paper for BAT in immediate drug hypersensitivity, it was weakly recommended to re‐test nonresponder patients after 6 months [19], as nonreleasers can convert into releasers (see below). In studies involving cellular tests, the number of nonresponders should be documented. Calculations, such as sensitivity and specificity, should clearly indicate whether the group of nonresponders was included or excluded.

### 2.4. Factors Contributing to Nonresponsiveness

#### 2.4.1. Background

**Figure 2:**
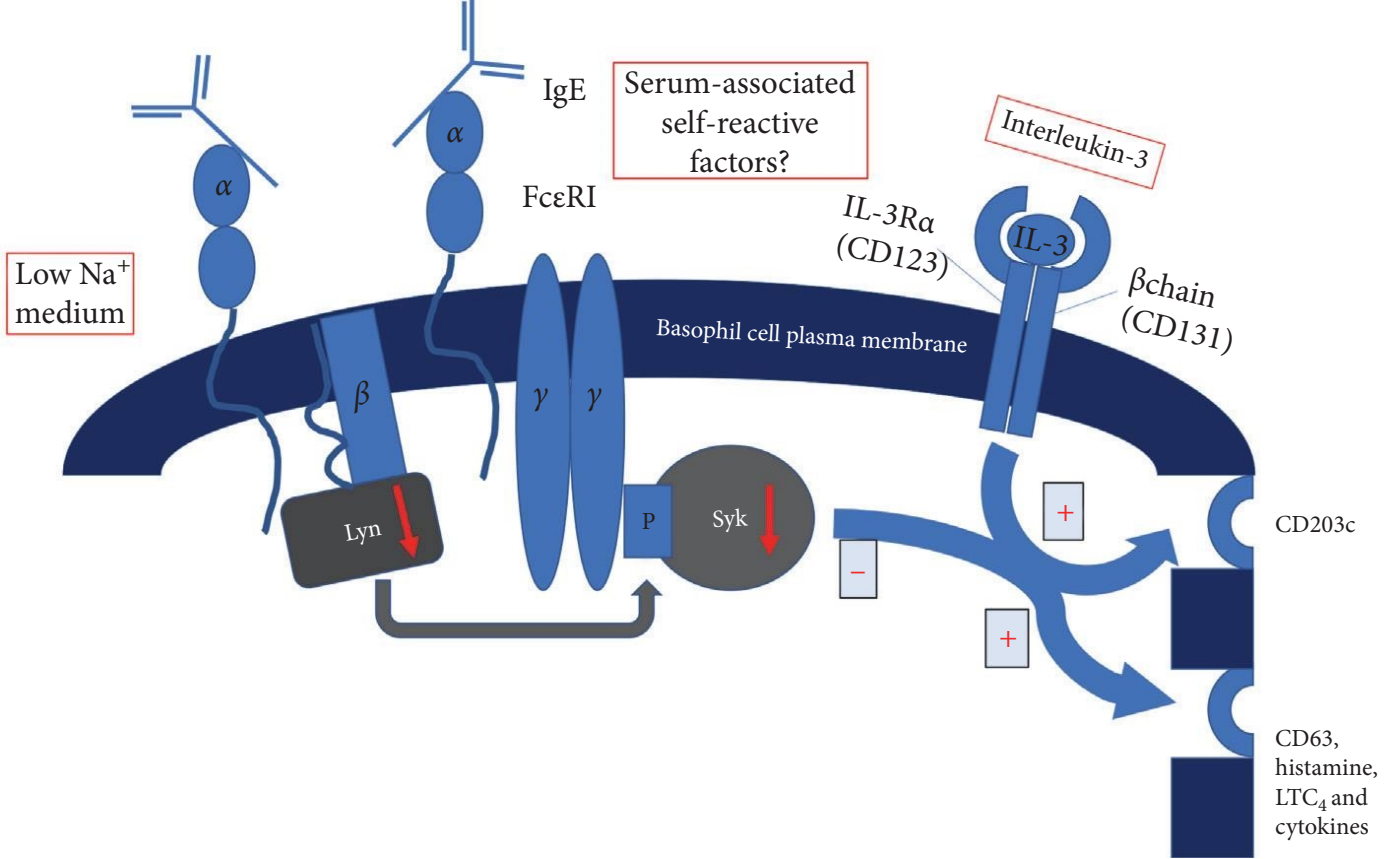
Simplified model for basophil activation by signaling through the FcɛRI and IL‐3 receptor in nonresponders. After FcεRI cross‐linking, reduced or absent levels of Lyn and Syk are observed, resulting in a lack of histamine release, upregulation of basophil activation markers, LTC_4_, and cytokines (modified after [21]). Prolonged incubation with IL‐3 and ionic factors can partially restore IgE‐dependent signal cascades. Serum‐associated self‐reactive factors might influence basophil reactivity.

The Fc part of IgE is bound to a receptor on the basophil cell plasma membrane. Studies of the interaction between IgE and Fc*ε*RI have shown that the interaction is of high affinity. The number of Fc*ε*RI per basophil varies between different donors and is related to the IgE concentration in the serum. Fc*ε*RI consists of four polypeptide chains, *α*βγ2, which together form an integral membrane protein complex with seven transmembrane segments. Of these domains *α*2 is directly involved in IgE binding, but maximal affinity is observed only when both domains are present. Together with the two *γ* subunits, the *β* subunit acts synergistically to promote signal transduction through the Fc*ε*RI. These chains are also involved in signal transduction from the receptors to the cell interior. The *β* and *γ* chains of the Fc*ε*RI complex contain immunoreceptor tyrosine‐based activation motifs (ITAMs). These have distinct functions. There are two species of Fc*ε*RI‐associated protein tyrosine kinases (PTKs) – the *src* family kinase Lyn and the p72Syk kinase. The former is found associated with Fc*ε*RI *β*, whereas the latter is capable of binding both Fc*ε*RI *β* and Fc*ε*RI *γ* but has a higher affinity for interaction with Fc*ε*RI *γ*. Cross‐linking of the Fc*ε*RI initiates the activation of *β*‐chain bound Lyn. Subsequently, Lyn phosphorylates the *β* and *γ*‐chain ITAMs. The phosphorylated *γ*‐chains then recruit the PTK‐Syk, which upon binding to the two phosphorylated ITAMs tyrosyls also undergoes activation. The activated Syk subsequently phosphorylates phospholipase C‐*γ*1 and phospholipase C‐*γ*2. These phosphorylated phospholipase C*γ*s catalyze the hydrolysis of the plasma membrane phosphatidylinositol 4,5‐bisphosphate, generating inositol 1,4,5‐trisphosphate and 1,2‐diacylglycerol. These second messengers promote the release of Ca^2+^ from internal stores and activate protein kinase C, respectively. Both events are essential for Fc*ε*RI‐mediated degranulation, which leads to the release of several mediators including histamine and leukotrienes [17]. In addition, activation markers like the granule‐associated molecule CD63 and the ecto‐enzyme CD203c are upregulated to the plasma membrane in response to Fc*ε*RI‐linkage. Based on time kinetics of upregulation, it was hypothesized that molecules of the “CD203c group” and the “CD63 group” were linked to two different mechanisms of basophil activation [20] (Figure 2 [21]).

#### 2.4.2. Genetic Factors

Gene expression in unstimulated nonreleaser basophils (<12.7% histamine release, *n* = 2) showed 59 genes transcribed at a lower level and 37 genes transcribed at a higher level compared to releaser donors. All molecules of interest (IgE receptor subunits, granule contents, kinase receptors, cytokines, chemokines, and IgE‐dependent histamine releasing factor), including Lyn, were expressed at lower levels in nonreleaser cells with except for TNF‐alpha‐induced protein 6, histamine receptor 4, and one serine/threonine kinase. After incubation with anti‐IgE, 85 genes were differentially expressed in these nonreleaser basophils, including two heat shock proteins, Lyn, and dual specificity phosphatase 6. In contrast to releaser cells, no chemokines or cytokines were found among the upregulated genes in activated nonreleaser basophils [8].

Another author stated, however, that expression profiling (mRNA signatures) of human basophils comparing releasers to nonreleasers (<1% histamine release) resulted in global variation within the expected range for little or no difference [7]. Gene array analysis in unstimulated basophils also detected no significant difference between reactive and anergic basophils. After Fc*ε*RI stimulation, no changes were observed in anergic basophils [14]. Another analysis showed that Syk mRNA was consistently present in basophils from five nonreleaser donors, although Syk protein levels were suppressed [22]. This was also found by another group showing equivalent levels of mRNA for Lyn and Syk kinase for both releasers and nonreleasers (<1% histamine release). By sequencing a critical region in the Syk mRNA, it was also demonstrated that the frame shift mutation in Syk leading to a premature stop codon, observed in other cell types, is not present in nonreleasing human basophils [23].

It was also mentioned that one limitation of these in vitro approaches is that simple isolation of basophils from circulation induces significant changes [7].

A trio of SNPs (rs290987, rs290988, and rs2562397) were identified in a group of 59 individuals as potential regulators of Syk expression, with the CC genotype of rs290988 being associated with poor histamine release (≤20% histamine release). If genetics were the primary determinant of Syk expression, then treatment with omalizumab should not affect Syk expression. However, treatment with omalizumab increased Syk expression in poor‐releasers, suggesting that the genetic regulation of Syk can be overcome [9].

SNP‐analysis in the IL‐3 gene with PCR amplification revealed in one study (*n* = 83 donors) that nonreleasers (<5% histamine release with ConA) showed the presence of SNP at + 79 (T‐C), leading to one amino acid change at the 8th position in the mature IL‐3 from serine to proline. The presence of this less potent isoform of IL‐3/P8 was suspected to be responsible for the nonreleaser phenotype [6].

In summary, there are indications of some genetic changes (SNPs), but these still need to be confirmed by further investigations.

#### 2.4.3. Ionic Regulation

Basophils from 12 out of 49 normal subjects, who did not release histamine when challenged with an optimal dose of anti‐IgE in a 135 mM NaCl buffer, were converted into releasing basophils when stimulated with anti‐IgE in a low‐Na^+^ medium. The increase in Na^+^ concentration in the extracellular medium led to a decrease in the basophil response to anti‐IgE, particularly in nonreleasers compared to releasers. As the Na^+^ concentrations increased, nonreleasers showed a progressive and almost complete inhibition of histamine release, unlike releasers [24]. Basophils with the nonreleasing phenotype exhibited an IgE‐mediated (Ca^++^)i response at the single‐cell level. However, the time lag for (Ca^++^)i in nonreleasing basophils was three times longer than in releasing basophils. Additionally, the (Ca^++^)i response was more asynchronous in nonreleasing basophils and lacked in a sustained (Ca^++^)i elevation compared to releasing basophils [25].

In summary, ionic regulation, specifically sodium (Na^+^), appears to be a significant factor influencing the ability of human basophils to respond to IgE‐mediated stimulation. However, it is unclear whether these effects also have a role in vivo.

#### 2.4.4. Conversion of Releasers Into Nonreleasers and Vice Versa Overtime

Some researchers have observed that over a period of up to 4 years, individuals who were originally nonreleasers can convert into releasers and vice versa [3, 14, 16]. However, not all individuals undergo this conversion process. For example, in one study 8 (out of 8) asthmatic nonreleasers and 16 (out of 23) nonasthmatic nonreleasers cycled to releaser status at least once over 4 years. Medication or season did not have an impact on this cycling. Additionally, the transition to nonreleaser basophils did not change the established hallmarks of asthma. Other researchers found that the shift from one nonreleaser to releaser status in an individual was associated with the presence of Lyn and Syk proteins in basophils at levels similar to those found in releaser basophils [16].

In summary, there are currently no clearly defined factors that can lead to the conversion from nonreleaser to releaser status, or vice versa, in vivo. However, they appear to be related to Syk expression.

#### 2.4.5. High‐Affinity IgE Receptor Fc*ε*RI

It has been known since 1973 that there are preparations of human basophils expressing normal levels of the high‐affinity IgE receptor Fc*ε*RI, but release little or no histamine in response to FC*ε*RI cross‐linking [26]. It was shown that in anti‐IgE‐nonreleasing basophils, anti‐IgE induces cross‐linking of the membrane‐bound IgE, but this did not lead to a rise in [Ca^++^]i [27]. Later, it was shown in three healthy nonatopic donors that nonreleasing basophils express Fc*ε*RI normally [16]. Also, no primary structural change of FC*ε*RI was observed in nonreleasing basophils (*n* = 7) [18].

This is in contrast to a flowcytometric analysis revealing a significantly reduced expression of Fc*ε*RI in nonreleasing basophils. In this study total serum IgE levels of 83 donors were also significantly low in nonreleasers as compared to releasers [28]. Another study revealed that “anergy” (with the critically viewed cut‐off point <38% CD63+ basophils) was associated with intermediate or low levels of IgE against house dust mite [14].

In summary, the Fc*ε*RI receptor does not appear to be structurally altered in nonreleasers, although its expression may be reduced in connection with low IgE levels. This requires further evaluation.

#### 2.4.6. Fc*ε*RI‐Associated PTKs (Lyn and Syk)

In 1999 it was shown through Western blotting that three nonatopic donors with nonreleaser basophils failed to express the protein for the tyrosine kinase Syk. Protein levels for the tyrosine kinase Lyn were somewhat reduced, but not absent [16]. Another group also reported reduced levels of Lyn protein in nonreleaser basophils (*n* = 2) compared to releaser basophils (*n* = 6). This group suggested that there may be translational or posttranslational regulatory mechanisms specific to the expression of these two important Fc*ε*RI‐associated signaling elements in basophils [23]. Flowcytometric analysis of nonreleaser basophils (*n* = 15) revealed a significantly reduced expression of Lyn and Syk kinases compared to releasers (*n* = 52) [6]. Similar results were found in another study with three nonreleaser and two releasers [16]. It was suggested that the loss of Syk protein in nonreleaser basophils is attributed in part to excess proteasome‐mediated degradation of Syk, as nonreleaser basophils treated with proteasome inhibitors restored Syk expression. Caspase and calpain inhibitors had only modest or no effects. It was also shown that ubiquitination of Syk was associated with a substantial decrease in total levels of Syk protein. Therefore, it was concluded that the signaling defect is primarily the result of excess Syk degradation [8, 29]. Others found in phosphorylation analysis that Syk downregulation was associated with the loss of Fc*ε*RI‐induced phosphorylation of kinases p38 and ERK1/2, which are kinases located in the signaling cascade downstream of Syk [14].

In summary, the most relevant factor appears to be a translational or posttranslational mechanism that is crucial for Syk degradation in nonresponders.

#### 2.4.7. Interleukin‐3

In 1996, it was demonstrated that the conversion of nonreleasing basophils (*n* = 3) to releasing basophils occurred after 3 days of culture (1 to 7 days) with 300 pmol/L IL‐3 [18]. Similarly, others were able to detect Syk expression after prolonged incubation with IL‐3 in nonreleasing basophils (*n* = 5) [22]. Therefore, it has been suggested that changes in serum IL‐3 levels could be a potential factor in the transition from nonreleasers to releasers. Measurements from five releasers and five nonreleasers showed values ranging from undetectable to 10 pg/mL, but did not show a clear correlation with releaser status [8]. Another group preactivated basophils with 10 ng/mL IL‐3 for 1 hr, 1, 2, 3 or 6 days before activation with allergen or anti‐IgE AB and measured CD203c‐upregulation. CD203c is already expressed at significant levels on resting basophils. In two nonresponders optimal CD203c upregulation was observed just 1 day after incubation with IL‐3. The authors suggested using an aliquot that can be stimulated overnight with IL‐3 and then used for conventional analysis the next day in nonresponders [30]. As mentioned earlier, the presence of a less potent isoform of IL‐3/P8 was suspected to be responsible for a nonreleaser phenotype [6].

Overall, there were 700–900 gene transcripts that changed significantly beyond a threshold derived from replicate variation (without adjustment for multiple tests) with or without IL‐3. The Benjamini–Hochberg FDR thresholds resulted in 229 and 100 changes, respectively for 10 ng/mL IL‐3 and no IL‐3. Comparing releasers to nonreleasers or atopics to nonatopics resulted in global variation that was within the range expected for little or no differences [7]. Additionally, it was demonstrated that stimulation of the IL‐3 receptor with IL‐3 led to similar phosphorylation levels of p‐ERK1/2, a kinase located in the signaling cascade downstream of Syk, in both reactive and anergic cells (with the critically viewed definition of having <38% CD63+ basophils to anti‐Fc*ε*RI) [14].

In summary, it can be said that interleukin‐3 appears to be an important modulating factor in vitro that can influence the Syk signaling cascade. However, in vivo, the role of this interleukin does not appear to be decisive.

#### 2.4.8. Other Factors

When nonresponding cells of seven patients were incubated with phorbol ester (phorbol 12‐myristate 13‐acetate) and challenged with either anti‐IgE or a specific antigen, histamine was released by nonresponding cells [31]. Modulating blood basophils with a selective inhibitor of the phosphatase function of SHIP‐1 (SH2‐containing inositol‐5′‐phosphatase 1) did not restore responsiveness to IgE stimulation in basophils from nonresponders (*n* = 6) [32]. The serum of three nonresponders was used in passive assays using progenitor‐derived basophils. A strong link between serum‐associated self‐reactive factors and the basophil nonresponder state in BAT was shown in vitro, but the clinical implications of this finding have yet to be determined [21].

In summary, both older and newer approaches were used to uncover mechanisms. However, they were either not pursued further or require additional investigations.

## 3. Clinical Implications and Alternative Assays

The IgE‐dependent nonresponse leads to false‐negative results in IgE‐mediated clinical reactions, meaning that the test does not provide any information about triggers and is uninterpretable. One can try adding interleukin‐3 or repeating the BAT after 6 months.

Other ways to overcome this limitation include the performance of passive tests with donor basophils, the aforementioned progenitor‐derived basophils [33] or humanized rat basophilic leukemia cells. These cells are passively sensitized with patients’ sera and activated by IgE‐dependent stimuli, but they have their limitations. Therefore, they are more commonly used for special research approaches.

Alternatively, mast cells can be used as another cell type that degranulates in response to IgE‐dependent stimuli. The simplest method is to perform a skin test (prick test or intracutaneous test) in vivo. In addition, several research groups have established mast cell activation tests (MAT) in vitro as a diagnostic tool for IgE‐mediated allergies, testing the reactivity of patients’ sera by passive sensitization. The MAT relies on flowcytometric quantification of activation and degranulation markers. Different kinds of mast cells have been used, such as mast cell lines, humanized mast cell progenitor lines and cultured primary human mast cells [34]. For hymenoptera venom allergy, MAT provided conclusive results in 20 of 37 patients (54.1%) with nonresponding basophils in the BAT [35].

## 4. Summary, Future Directions, and Research Opportunities

First, a uniform definition of nonresponsiveness should be established, considering the results of previous studies, established thresholds, expert opinions, and practical aspects.

Regarding the underlying mechanisms, the implicit hypothesis based on the studies presented is that the nonresponder status is a result of reduced Lyn/Syk kinase expression or posttranslational regulation (proteasomal degradation), which can sometimes be overcome by IL‐3 or changes in ion levels in vitro. Alternative explanations could include basophil heterogeneity, receptor polymorphisms or effects of systemic immune regulation. Nonresponder status does not seem to be a permanently fixed phenotype but rather appears to be modifiable by factors that are not yet fully understood, at least in vivo.

The problems with the studies presented here include the small number of individuals examined in some cases, their heterogeneous nature, as well as the fact that their results are correlative rather than causal. Furthermore, the prevalence data varies, some mechanisms are anecdotal, evaluations are inconsistent (particularly with regard to flow cytometry gating), and there is a lack of quantitative synthesis of effect sizes. Therefore, further prospective and larger studies, including multicenter interlaboratory studies are needed, to demonstrate the reproducibility of the results. Additionally, more longitudinal studies should be conducted to track dynamic transitions between responders and nonresponders over time. Further additional biochemical studies with mechanistic experiments (such as Lyn/Syk knockdown/overexpression studies) and multiomics approaches (including proteomics, phosphoproteomics, and single‐cell RNA‐sequencing) are necessary to identify consistent features and explore the natural in vivo factors that influence the transition from being a nonresponder to a responder, and vice versa. The focus should be on mechanistic studies of Lyn/Syk regulation. Furthermore, passive tests should be more widely established in clinical practice to combat the issue of false‐negative results in basophils.

## Ethics Statement

The authors have nothing to report.

## Consent

The authors have nothing to report.

## Conflicts of Interest

Bernadette Eberlein reports financial support from BÜHLMANN Laboratories outside the submitted work.

## Author Contributions

Bernadette Eberlein wrote the manuscript and Tilo Biedermann reviewed the manuscript.

## Funding

Open Access funding enabled and organized by Projekt DEAL.

## Data Availability

Data sharing is not applicable to this article as no new data were created or analyzed in this study.
